# Tissue- and hormone- dependent progesterone receptor distribution in the rat uterus

**DOI:** 10.1186/1477-7827-4-47

**Published:** 2006-09-11

**Authors:** Lena Sahlin, Britt Masironi, Sonja Åkerberg, Håkan Eriksson

**Affiliations:** 1 Division for Reproductive Endocrinology, Department of Woman and Child Health, Karolinska Institutet, Stockholm, Sweden

## Abstract

**Background:**

Estradiol (E2) and progesterone (P) are well known regulators of progesterone receptor (PR) expression in the rat uterus. However, it is not known which receptor subtypes are involved. Little knowledge exist about possible differences in PR regulation through ERalpha or ERbeta, and whether the PR subtypes are differently regulated depending on ER type bound. Thus, in the present study PR immunostaining has been examined in uteri of ovariectomized (ovx) rats after different treatments of estrogen and P, in comparison with that in immature, cycling, and pregnant animals.

**Methods:**

The uteri were collected from 1) ovx rats treated with E2 and/or P; 2) immature rats, intact cycling rats and animals pregnant day 8 and 18; 3) ovx rats treated with E2 or an estrogen receptor (ER)alpha agonist or an ERbeta agonist. Two antibodies were used, one detecting PRA+B and another one specific for PRB. Real-time PCR was used to determine mRNA levels for PRAB and PRB in experiment 3.

**Results:**

In stroma and myometrium faint staining was detected in ovx controls (OvxC), whereas E2 treatment resulted in strong staining. In contrast to this, in luminal epithelium (LE) the staining was strong in the OvxC group, whereas E2 treatment during the last 24 hrs before sacrifice caused a decrease. Similar to OvxC the LE of the immature animals was strongly stained. In the pregnant rats LE was negative, well in agreement with the results seen after E2 treatment. In the pregnant animals the stroma and decidua was strongly stained for PRAB, but only faint for PRB, indicating that PRA is the most expressed isoform in this state. The increase in stromal and myometrial immunostaining after E2 treatment was also found after treatment with the ERalpha agonist PPT. The ERbeta agonist DPN caused a decrease of the PR mRNA levels, which was also found for PRAB and PRB immunostaining in the GE.

**Conclusion:**

Stromal and myometrial PRAB levels are increased via ERalpha, as shown by treatment with E2 and the ERalpha agonist PPT, while the levels in LE are decreased. The uterine stroma of pregnant rats strongly expressed PRAB, but very little PRB, which is different to E2 treated ovx animals where both PRAB and PRB are strongly expressed. The ERbeta agonist DPN decreased the mRNA levels of PRAB and PRB, as well as the PRAB protein level in GE. These results suggest that ERbeta signals mainly down-regulate PR levels in the epithelial cells. ERalpha, on the other hand, up-regulates PR levels in the stroma and myometrium while it decreased them in LE. Thus, the effects from E2 and PPT on the mRNA levels, as determined by PCR, could be annihilated since they are increased and decreased depending on cell type. The distribution and amount of PR isoforms strongly depend on the hormonal milieu and cell type within the rat uterus.

## Background

Progesterone (P) together with estrogen provides the basis for the cyclic changes in the uterine tissues during the estrous cycle. Stromal-epithelial interactions have been shown to be critical in the regulation of epithelial cells by estradiol (E2) and P [1]. The actions of E2 and P are primarily mediated via binding to specific intracellular receptors in the target cells. The estrogen receptor (ER) and progesterone receptor (PR) are members of a superfamily of nuclear transcription factors with highly homologous DNA binding and ligand binding domains [2-6].

PR exists in two major isoforms, A and B [7]. The two isoforms arise due to use of different promoters, thus creating two separate mRNAs. It has been shown that PR is localized in the nuclei of epithelial, stromal and smooth muscle cells in the uterus of normal cycling rats [8,9]. In addition, estrogens increase the PR immunoreaction in stromal, but not epithelial, cells in ovariectomized (ovx) rats. Thus, these results made Ohta et al. conclude that uterine PR expression is regulated by ovarian steroids during the estrous cycle and early pregnancy [8].

After the discovery of ER subtype (β) [2], the hormonal signals are now assumed to be transduced by both forms of ER, α and β [2-5]. Both ERs bind E2 with high affinity and specificity [10]. Although ERβ shares many functional characteristics with ERα, the molecular mechanisms regulating the transcriptional activity and the tissue location of ERβ are distinct from those of ERα [2,10].

In ovx rats, E2 induces DNA synthesis and mitosis in the uterus, whereas P inhibits DNA synthesis in the epithelium, but stimulates mitosis in the stromal cells [11,12]. ERα turns on target gene expression and functions as a regulator of ligand-activated transcription in estrogen responsive tissues [13], whereas P attenuates cell sensitivity to E2 by decreasing ERα levels [14]. It has been shown that nuclear ERα levels decrease in the rat uterus as serum P levels increase [15], and that P decreases sensitivity of cells to estrogens by inhibiting ER-mediated transactivation via direct interactions of ligand bound PR and ERα [16].

The aim of the present study was to determine the distribution of PR, also regarding subtype, in the rat uterus during different hormonal conditions. We also aimed at detecting possible differences between stimulation by ERα and ERβ. We have studied PRAB and PRB immunostaining in the uteri of ovx rats treated with an ERα agonist, an ERβ agonist, E2 and/or P and for comparison the results from immunostaining of immature animals, intact cycling animals and during pregnancy is also shown.

## Methods

### Animals

All rats were purchased from Scanbur-BK AB, Sollentuna, Sweden. The animals were housed in a controlled environment at 20°C on an illumination schedule of 12 h of light and 12 h of darkness each day. Standard pellet food and water were provided ad libitum. The animal studies were approved by the Committee on Animal Care in Sweden.

*Experiment 1*. Forty-one adult female Sprague-Dawley rats, 55–60 days old, were ovariectomized under light ether anaesthesia and housed for 14 days before initiation of hormone treatment. They were treated with 1 μg 17β-estradiol (E2)/100 g bw and/or 0.4 mg progesterone (P)/100 g bw as shown in Table 1. The designation of the treatment groups as described in Table 1 will be used in the text.

**Table 1:** Experiment 1

**Experimental Group**	**Day 1 (8 am) treatment**	**Day 2 (8 am) treatment**	**Day 3 (8 am)**	**Uterine weight (g)**
**OvxC **(n = 5)	vehicle	vehicle	sacrifice	0.092^a ^± 0.008
**24E **(n = 6)	E2	sacrifice		0.162^bcd^* ± 0.019
**24P **(n = 6)	P	sacrifice		0.122^abc ^± 0.013
**48E **(n = 6)	E2	E2	sacrifice	0.215^b^* ± 0.039
**48P **(n = 6)	P	P	sacrifice	0.098^ad ^± 0.008
**24E+24P **(n = 6)	E2	P	sacrifice	0.145^abc ^± 0.012
**24P+24E **(n = 6)	P	E2	sacrifice	0.170^c^* ± 0.018

The rats were injected s.c. in the neck with either vehicle (propyleneglycol), 10 μg E_2_/kg rat, or 4 mg P_4_/kg rat, or combinations of these. Experimental animals were sacrificed after 24 or 48 hours of hormone treatment, respectively. The uterine wet weight is presented in grams (mean ± SD). Numbers (right column) without any common superscript letters indicate a significant (p < 0.05) difference in uterine weight. The asterisk (*) indicates a significant (p < 0.05) difference in uterine weight as compared to OvxC.

*Experiment 2*. Four groups of 6 rats each, see Table 2 for a more precise description of the rats. The pregnant rats (n = 12) arrived at the animal department on day 4 of pregnancy together with their non-pregnant littermates (n = 6). The pregnant rats were sacrificed on day 8 (n = 6) and 18 (n = 6) of pregnancy. Group I: Immature 21-days old. Group II: Intact cycling rats 8 weeks old, vaginal smear was take n at sacrifice to determine stage of estrous cycle. Group III: 8-days pregnant rats. From group III one rat was deleted due to lack of embryos, i.e. it was not pregnant. Group IV: 18-days pregnant rats. From group IV one rat was deleted since it carried only one embryo, which was very different to the others who carried between 9–13 embryos each. Group III and IV therefore have n = 5.

**Table 2:** Experiment 2

**Experimental group**	**Uterine weight (g)**	**Number of pups**
Immature (n = 6)	0.03 ± 0.00^a^	-
NC^§ ^(n = 6)	0.29 ± 0.059^ab^	-
Pregnant day 8 (n = 5)	0.57 ± 0.062^#bc^	11–15
Pregnant day 18 (n = 5)	25.8 ± 4.06^#c^	9–13

^§ ^NC = normal cycling controls, three in metestrus, two in proestrus and one in diestrus

^# ^including placenta

Uterine weights and number of pups in the different groups. The uterine wet weight is presented in grams (mean ± SD). Numbers without any common superscript letters are significantly different in uterine weights.

**Table 3:** Immunostaining results from the rats in experiment 2

**Experimental Group ***(rat number)*	**PRAB**				**PRB**			
**Immature ***(n = 6)*	LE	GE	Stroma	Myomet	LE	GE	Stroma	Myomet
1	+++	0	+	+	+++	0	+	+
2 na								
3 na								
4	+++	+++	+	(+)	+++	++	+	+
5 na								
6	+++	+++	+	(+)	+++	++	+	+

**NC ***(n = 6)*								
1 (metestrus)	-	+	(+)	+	lost	lost	lost	lost
2 (proestrus)	+	+	+	++	+	(+)	(+)	+
3 (metestrus)	-	-	+	++	+	+	+	+
4 (late proest)	-	+	+++	+++	-	-	+	+++
5 (diestrus)	+++	+	++	++	+++	++	++	++
6 (metestrus)	(+)	+	(+)	++	+	+	(+)	+

**Pregnant day 8***(n = 5)*				Decidua				Decidua
1	-	-	+++	+++	-	-	(+)	+
2	-	-	+++	+++	-	-	(+)	+
3	-	-	+++	+++	-	-	(+)	+
4	-	-	+++	+++	-	-	(+)	+
5	-	-	++	+++	-	-	(+)	+

**Pregnant day 18 ***(n = 5)*			Placenta	Decidua			Placenta	Decidua
1			-	+++			-	+
2			-	+++			-	+
3			-	+++			-	+
4			-	+++			-	+
5			-	+++			-	+

na = not available 0 = no gland – = no staining

*Experiment 3*. Thirty-two female adult Sprague-Dawley rats, 55–60 days old and weighing approximately 250 g were used. The rats were ovariectomized during anesthesia with Xylazin (Bayer AG, Leverkusen, Germany; 0.75 mg/100 g rat) and Ketaminol (Intervet AB, Boxmeer, Netherlands; 7 mg/100 g rat). They were housed for 14 days before initiation of hormone treatment. The ovx animals were treated with one single s.c. injection with 2 μg E2/100 g bw, 0.5 mg PPT (4,4',4"-(4-Propyl- [1*H*]-pyrazole-1,3,5-triyl)*tris*phenol)/100 g bw or 1.25 mg DPN (2,3-*bis*(4-hydroxy-phenyl)-propionitrile)/100 g bw 18 hrs prior to sacrifice. The uterine weight of the PPT treated animals was higher than for the other three groups (p < 0.05), and the uterine weight of the E2 treated rats was higher than for the OvxC and DPN groups (p < 0.05; data not shown). Thus, the estrogenic effect of PPT binding to ERα was even stronger than that of E2 in this experiment, while DPN had no effect on uterine weight.

The non-steroidal compounds PPT and DPN were recently developed and characterized as selective agonists for ERα and ERβ, respectively. PPT is approximately 1000-fold more potent as an agonist on ERα than on ERβ and has a 400-fold preference towards ERα in its binding affinity [17,18]. DPN has a 70-fold higher relative binding affinity and 170-fold higher relative potency in transcription assays with ERβ than with ERα [19]. 17β-estradiol has equal affinity for ERα and ERβ [10]

### Hormones

E2 and P were purchased from Sigma Co. (St. Louis, Missouri). The hormones were dissolved in 99.5% ethanol at a high concentration and then diluted in propyleneglycol to the proper concentration. The final concentration of ethanol was less than 5% in the injections. The substances were injected in 200 μl. The OvxC group received vehicle (propyleneglycol).

For experiment 3 E2 was dissolved in 99.5% ethanol at a high concentration and then diluted with 50:50 DMSO:PBS to the proper concentration. The final concentration of ethanol was less than 2% in the injections. PPT and DPN were bought from Tocris Cookson, via Bio Nuclear, Bromma, Sweden, and were dissolved in DMSO and then diluted with PBS until the proper concentration and a dilution of 50:50 DMSO:PBS. The control group was treated with the vehicle (DMSO:PBS 50:50).

### Tissue collection

During anesthesia, the rat uterus was removed, stripped of fat and connective tissue, weighed and immersion-fixed in 4% formaldehyde for 8 hours and stored in 70% ethanol at 4°C and thereafter embedded in paraffin. In experiment 3 a small piece of uterus was also directly frozen in RNAlater (Ambion) and stored at -20°C until RNA preparation.

### Immunohistochemistry

Paraffin sections (5 μm) were used and a standard immunohistochemical technique (avidin-biotin-peroxidase) was utilized as described before [20], to visualize PRAB and PRB immunostaining intensity and distribution. There were 2–4 sections from every uteri/slide and there was always a positive and negative control included for every assay. When possible all samples in an experiment are run in the same assay to minimize variability due to technical handling. Only experiment 1 had to be divided into two separate assays, and then half of the slides in each group were run in each run to avoid any systematic error.

Since PRA is basically impossible to detect by immunohistochemistry, we have used one antibody which detect only PRB and one that binds both PRA and PRB. The differences in results obtained by the two antibodies should though be interpreted with caution, since it is difficult to draw conclusions on the expression of PRA when the affinities of each antibody are not the same. There is an antibody detecting human PRA available (Novacastra, clone 16, binds PRA in immunohistochemistry and PRA+PRB in Western blot)[21], but it does not work in rat tissue (data not shown). Monoclonal mouse anti-human antibodies were used for detection of PRAB (MA1-410, Affinity Bioreagents Inc.) and PRB (MA1-411, Affinity Bioreagents Inc.). The primary antibody was replaced by normal mouse IgG to obtain negative controls. ThePRAB antibody was diluted 1:100 in PB S and incubated on sections at 4°C overnight. The PRB antibody was diluted 1:100 in PBS, and incubated on sections at RT for 60 min. Following primary antibody binding, the sections were incubated with the second antibody, a biotinylated horse anti-mouse IgG (Vectastain, Vector, Laboratories, CA) diluted in normal horse serum, for 30 min at RT. Thereafter the tissue sections with PRAB were incubated for 30 minutes and with PRB for 60 minutes at RT with a horseradish peroxidase-avidin biotin complex (Vectastain Elite, Vector, Laboratories, CA). The site of the bound enzyme was visualized by the application of 3,3'-diaminobenzidine (DAB kit, Vector, CA), a chromogen, which produces a brown, insoluble precipitate when incubated with enzyme. The sections were counterstained with haematoxylin and dehydrated before they were mounted with Pertex (Histolab, Gothenburg).

### Manual scoring

One observer blinded to the identity of the slides, performed the assessment twice for the IHC scoring of experiment 1 and 2. The staining was evaluated semi-quantitatively using a grading system. The staining intensity was graded on a scale of (0) = no staining, (1) faint staining, (2) moderate staining and (3) strong staining.

### Image analysis

A Leica microscope connected to a computer using Colorvision software (Leica Imaging System Ltd. Cambridge, England) was used to assess immunostaining quantitatively by a computer image analysis system. Quantification of immunostaining was performed on the digitized images of systematic randomly selected fields of endometrial stroma, from which non-stromal elements (e.g. luminal epithelium, glandular epithelium and myometrium) were interactively removed and analyzed separately. All luminal epithelia, all glands, as well as 10 fields of stromal cells and 10 fields of myometrium were measured separately in each tissue section. By using colour discrimination software, the total area of positively stained cells (brown reaction product) was measured, and expressed as a ratio of the total area of cell nuclei (brown reaction product + blue haematoxylin).

### RNA isolation

Uterine tissue was placed in a RNA stabilization solution (RNAlater^®^, Ambion, Austin, TX) immediately after collection and stored at -20°C. Total RNA from 20 mg uterine tissue from each animal was purified with the RNeasy^® ^kit (Qiagen GmbH, Hilden, Germany) according to a procedure recommended by the manufacturer for RNA isolation from fibrous tissues.

### Reverse transcription

Two μg of total RNA from each sample was reverse transcribed at 37°C for 60 min in a final volume of 20 μl with a reaction mixture (Qiagen GmbH, Hilden, Germany) containing 1 × RT buffer, dNTP mix (0.5 mM each dNTP), 300 ng random primers (Invitrogen, Paisley, UK), 10 units RNase inhibitor (Superase-In, Ambion, Austin, TX), and 4U of Omniscript™ reverse transcriptase (Qiagen).

### Real time PCR for PRAB, PRB and RPLP0

Real time PCR was performed in a DNA Engine Opticon™ 2 System (MJ Research, Waltham, MA). For PCR, the cDNAs corresponding to 100 ng RNA were added to 10 μl of Quantitect™ SYBR^® ^Green PCR mix (Qiagen) containing HotStarTaq DNA polymerase, PCR buffer, dNTP mixture and 0.3 μM of each oligonucleotide primer in a final volume of 20 μl. The reactions were performed in opaque white 0.2 ml low-profile strip tubes sealed with optical flat caps (TLS-0851, TCS-0803, MJ Research, Waltham, MA). After initial incubation for 15 min at 95°C, the samples were subjected to 40 cycles of 10s at 94°C, 15s at 60°C (RPLP0 (ribosomal protein, large, P0) 57°C) and 20s at 72°C with a final extension step at 72°C for 5 min. All reactions were performed twice. The amount of PCR products for PRAB, PRB and RPLP0 increased linearly up to 24, 25 and 21 cycles, respectively. The purity of PCR products was confirmed by a melting curve analysis in all experiments (data not shown). The oligonucleotide primers for PRAB, PRB and RPLP0 are listed in Table 4. All primers were designed to span an intron/exon boundary or to flank an intron, thus, amplification of contaminating DNA was eliminated. Each PCR assay included a negative control containing a RNA sample without reverse transcription.

**Table 4:** Oligonucleotide primers used for real-time PCR in experiment 3.

**Gene**	**Accession No.**	**Primers**	**Position**
PRAB	NM_022847	Forward CAGGCCGCGGTGCTCAA	bp 1546–1572
		Reverse GTGGGCTCTGGCTGGCTTCT	bp 1636–1617
			Product 91 bp
PRB	U06637	Forward CAGACCAACCTGCAACCAGAA	bp 1471–1491
		Reverse AGTCCTCACCAAAACCCTGGG	bp 1592–1572
			Product 122 bp
RPLP0	NM_001002	Forward GGCGACCTGGAAGTCCAACT	bp 195–214
		Reverse CCATCAGCACCACAGCCTTC	bp 343–324
			Product 149 bp

### Quantification of mRNA

To standardize the quantification method, RPLP0 was selected out of several tested housekeeping genes as an invariable internal control. The PCR amplification rate and the cycle threshold (Ct) values were related to a standard curve using Opticon Monitor 3.0 software (MJ Research, Waltham, MA). The values of relative expression of genes of interest were normalized against the RPLP0 product.

### Statistics

Statistical calculations on the results from the hormone treated animals were performed by ANOVA on ranks (Kruskal-Wallis test) followed by Dunn's test for evaluation of significance. The results are presented as box and whisker plots. Values with an asterisk are significantly different (p < 0.05) to OvxC.

## Results

### Estradiol and progesterone treatment (experiment 1)

**Figure 1:**
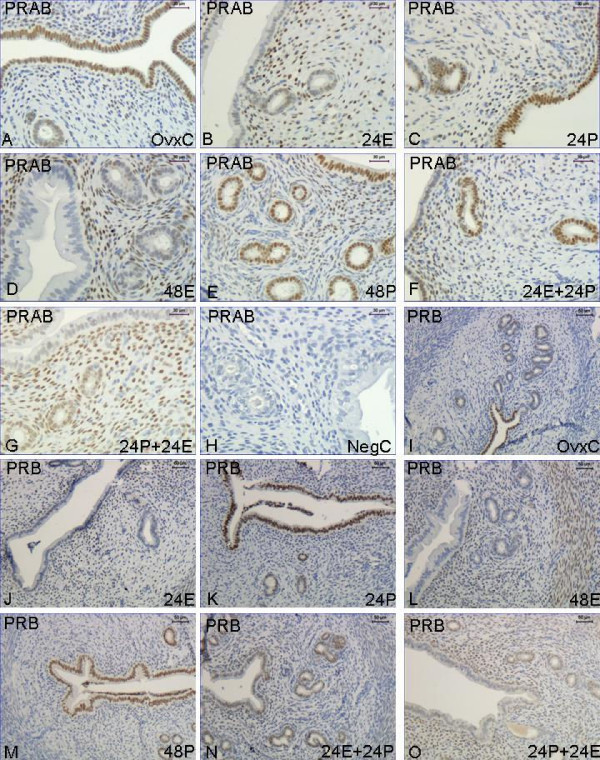
**Immunohistochemistry experiment 1**. Representative images of immunohistochemical results from each hormone treatment group in experiment 1, PRAB **A-H**, PRB **I-O**. Treatment groups are as follows: OvxC: **A **and **I**, 24E: **B **and **J**. 24P: **C **and **K**, 48E: **D **and **L**, 48P: **E **and **M**, 24E+24P: **F **and **N**, 24P+24E: **G **and **O**. Magnification bar 30 μm in images A-H and 50 μm in images I-O.

**Figure 2:**
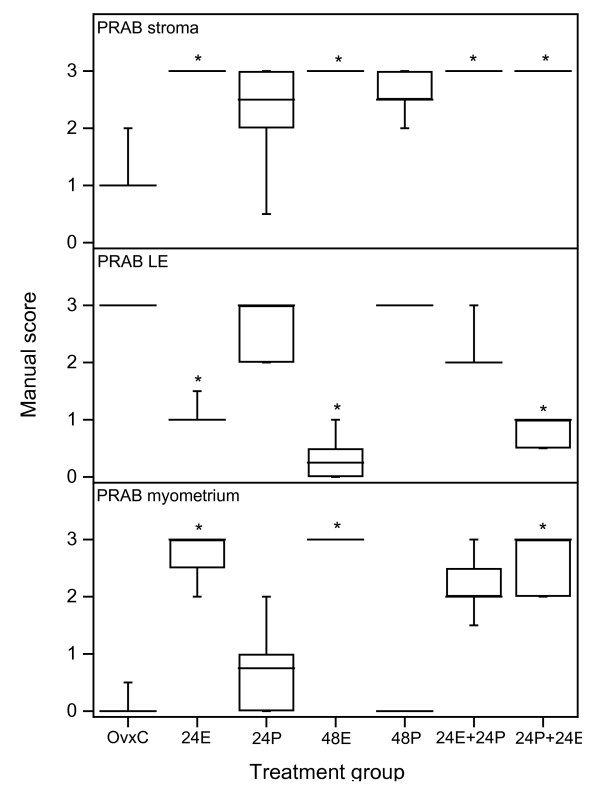
**Scoring results PRAB experiment 1**. Results from manual scoring of PRAB immunohistochemistry results in stroma (upper panel), luminal epithelium (LE, middle panel) and myometrium (bottom panel). The "box-and-whisker plot" represents the median value with 50% of all data falling within the box. The whiskers extend to the 5th and 95th percentiles. An asterisk indicates a significant (p < 0.05) difference compared to the OvxC group.

*PRAB*. Immunostaining in the stroma was faint in the OvxC rats (Fig 1A), but was significantly increased in all groups receiving E2 at any time (Fig. 2, top panel, Fig 1B,D,F,G). The values showed a tendency to increase in groups 24P and 48P but did not reach significant levels (Fig. 2, top panel, Fig 1C,E). In LE staining was strong in the OvxC (Fig 1A), but decreased in all groups that were treated with E2 the last 24 hrs before sacrifice (Fig 2, middle panel; Fig 1B,D,G). The staining of LE in the rats of group 24P, 48P and 24E+24P did not differ compared with OvxC (Fig 2, middle panel; Fig 1C,E,F). There was no staining in the myometrium of the OvxC group, but there was strong staining in all groups receiving E2 the last 24 hrs before sacrifice (Fig 2, bottom panel). The groups treated with P the last 24 hrs before sacrifice did not differ in immunostaining of the myometrium compared with the OvxC group (Fig 2, bottom panel). None of the treatment groups were different from OvxC in PR expression of the GE (data not shown).

**Figure 3:**
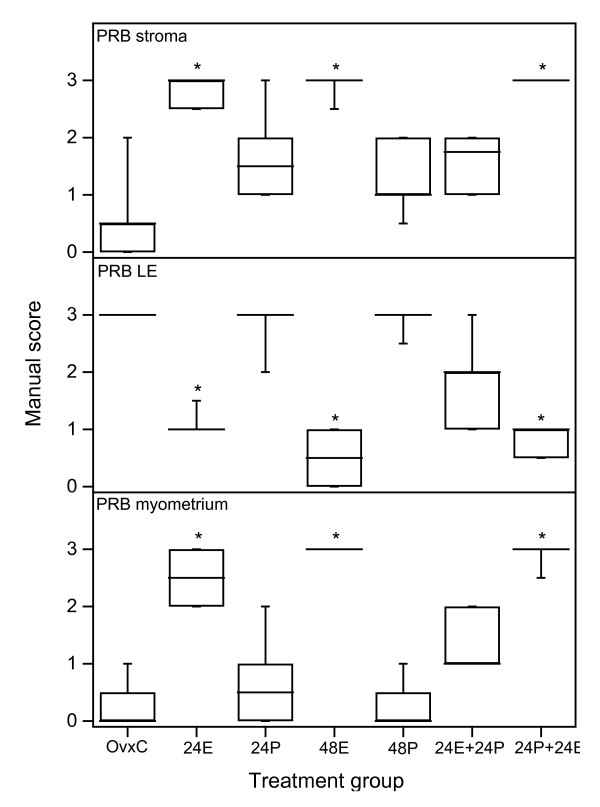
**Scoring results PRB experiment 1**. Results from manual scoring of PRB immunohistochemistry results in stroma (upper panel), luminal epithelium (LE, middle panel) and myometrium (bottom panel). The "box-and-whisker plot" represents the median value with 50% of all data falling within the box. The whiskers extend to the 5th and 95th percentiles. An asterisk indicates a significant (p < 0.05) difference compared to the OvxC group.

*PRB*. There was faint staining in the uterine stroma of the OvxC group (Fig 1I), but E2 treatment the last 24 hrs before sacrifice resulted in strong staining (Fig. 3, top panel; Fig 1J,L,O). P treatment the last 24 hrs before sacrifice showed an intermediary type of staining, but it was not different from either OvxC or the E2 treated animals (Fig 3, top panel; Fig. 1K,M,N). The PRB immunostaining in the LE of the OvxC group was very strong (Fig 1I), but was decreased in the groups that received E2 the last 24 hrs before sacrifice (Fig 3, middle panel; Fig 1J,L,O). The 24P and 48P groups showed a similar strong staining as the controls (Fig 1K,M), whereas the 24E+24P group showed an intermediary type of staining (Fig 1N), not significantly different from either OvxC or E2 treated groups (Fig 3, middle panel). There was very faint staining in the myometrium of the OvxC group (Fig 3, bottom panel, Fig 1I). E2 treatment during the last 24 hrs prior to sacrifice resulted in very strong immunostaining of the myometrium (Fig 3, bottom panel; Fig 1J,L,O). In the GE none of the treatment groups were different to the OvxC group (data not shown). Normal mouse IgG replaced the primary antibodies to obtain negative controls for the immunohistochemistry assays, the result was completely negative (Fig 1H).

### Pregnancy (experiment 2)

**Figure 4:**
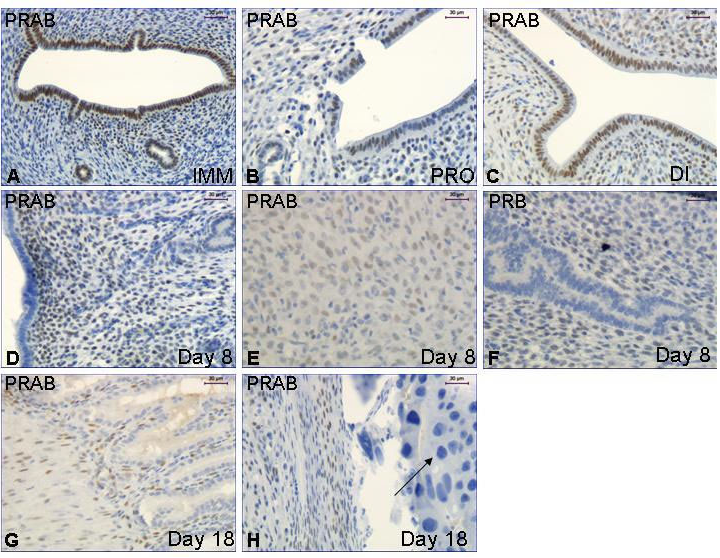
**Immunohistochemistry experiment 2**. Representative images of immunohistochemical results from the immature (**A**), cycling (**B,C**) and pregnant rats (**D-H**). Image F shows PRB immunostaining while all the others show PRAB. The arrow in image H indicates the negative cells in the placenta. Magnification bar 30 μm in all images.

*PRAB*. There was strong immunostaining in LE and GE of the immature animals, whereas it was faint in stroma and myometrium (Fig. 4A, Table 3). In the cycling intact controls (8 weeks old) there was faint staining in the LE and GE of the rats in proestrus (Fig. 4B) and moderate staining in the LE of a rat in diestrus (Fig. 4C). In stroma there was faint staining in all rats but the one in late proestrus, which was strong (Table 3). The staining of the myometrium was moderate in most cycling animals (Table 3). The stroma and decidua was strongly stained in the animals pregnant at day 8 (Table 3, Fig 4D,E), while LE and GE were negative (Fig. 4D, Table 3). The placenta was negative, but the decidua still strongly stained, in the rats pregnant on day 18 (Table 3, Fig. 4G,H).

*PRB*. There was strong PRB immuno-staining in the LE but moderate in the GE of the immature rats (Table 3), and there was faint staining in the stroma and myometrium (Table 3). In the cycling animals there was faint or no staining in LE, GE and stroma of all animals but one in diestrus. In the myometrium of the rat in late proestrus the staining was strong while the others were faint, or moderate (Table 3). In early pregnancy the LE (Fig 4F) and GE were negative, while the decidua was faintly stained (Table 3). In the late pregnant group the placenta was negative and the deciduas still faintly stained (Table 3).

### Estrogen receptor agonists (experiment 3)

**Figure 5:**
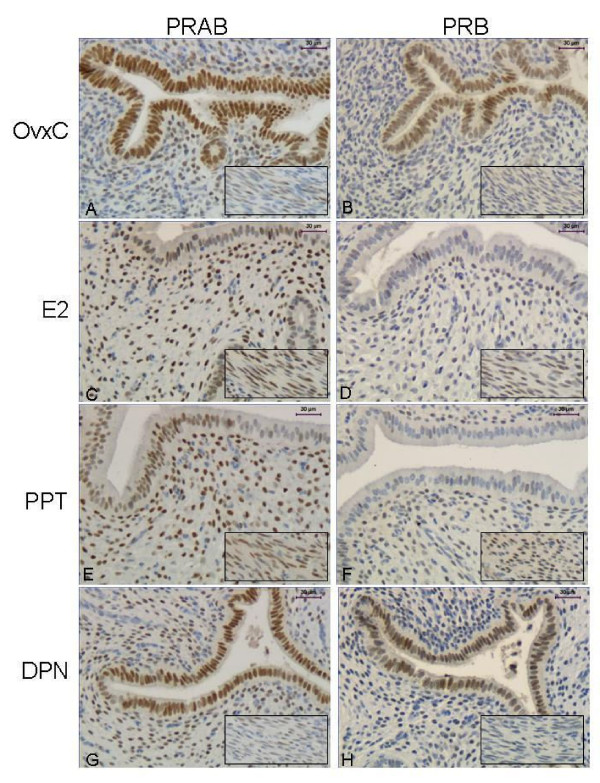
**Immunohistochemistry experiment 3**. Representative images of immunohistochemical results from the agonist treated rats, PRAB in left columnand PRB in right column. Representative images are shown from a rat in each treatment group as follows: OvxC (**A,B**), E2 (**C,D**), PPT (**E,F**) and DPN (**G,H**). The insert shows the myometrium from the same uterus. Magnification bar 30 μm.

**Figure 6:**
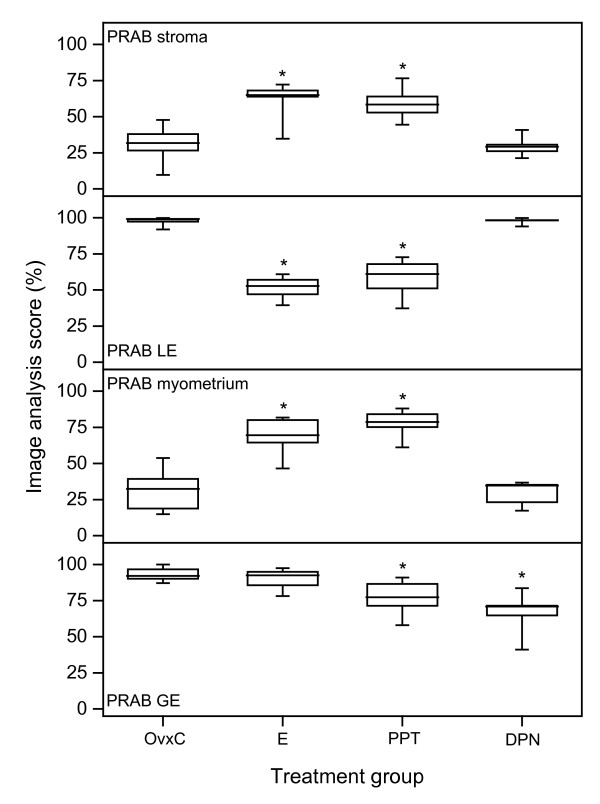
**Image analysis PRAB experiment 3**. Results from image analysis of PRAB immunohistochemistry results in stroma (upper panel), luminal epithelium (LE, second panel), myometrium (third panel) and glandular epithelium (GE, bottom panel). The "box-and-whisker plot" represents the median value with 50% of all data falling within the box. The whiskers extend to the 5th and 95th percentiles. An asterisk indicates a significant (p < 0.05) difference compared to the OvxC group.

*PRAB*. Stromal immunostaining increased after E2 and PPT treatment, while no effect was found in the DPN group (Fig. 5A,C,E,G; Fig 6 top panel). In LE E2 and PPT treatment resulted in decreased PRAB immunostaining, from nearly 100% to around 60%, while DPN had no effect (Fig. 5A,C,E,G; Fig 6 second panel). In the myometrium there was faint staining in the OvxC group, E2 and PPT treatments increased the PRAB staining while DPN had no effect (inserts in Fig. 5A,C,E,G; Fig. 6 third panel). In GE both PPT and DPN decreased immunostaining as compared to OvxC (Fig. 6, bottom panel).

**Figure 7:**
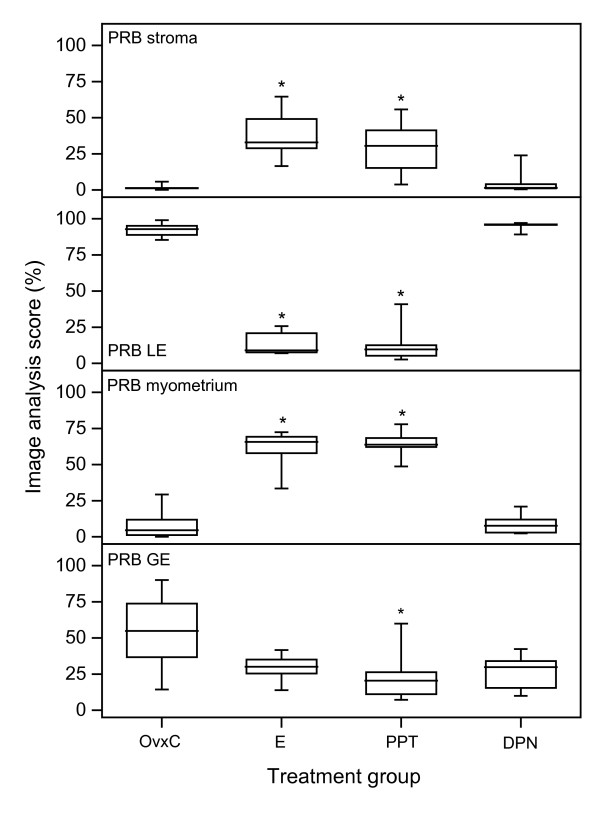
**Image analysis PRB experiment 3**. Results from image analysis of PRB immunohistochemistry results in stroma (upper panel), luminal epithelium (LE, second panel), myometrium (third panel) and glandular epithelium (GE, bottom panel). The "box-and-whisker plot" represents the median value with 50% of all data falling within the box. The whiskers extend to the 5th and 95th percentiles. An asterisk indicates a significant (p < 0.05) difference compared to the OvxC group.

*PRB*. Stromal immunostaining increased after E2 and PPT treatment, while no effect was found in the DPN group (Fig 5B,D,F,H; Fig 7 top panel). In LE E2 and PPT treatments resulted in a major decrease of PRB immunostaining, from over 90% to below 20%, while DPN had no effect (Fig 5B,D,F,H; Fig 7 second panel). In the myometrium E2 and PPT treatments increased the PRB immunostaining compared with the OvxC group and after DPN treatment (inserts in Fig 5B,D,F,H; Fig. 7 third panel). PPT decreased immunostaining in GE, as compared to OvxC (Fig. 7, bottom panel).

Normal mouse IgG replaced the primary antibodies to obtain negative controls for the immunohistochemistry assays, the result was completely negative (data from this experiment not shown; the results were similar to Fig 1H).

### PR mRNA expression in (experiment 3)

**Figure 8:**
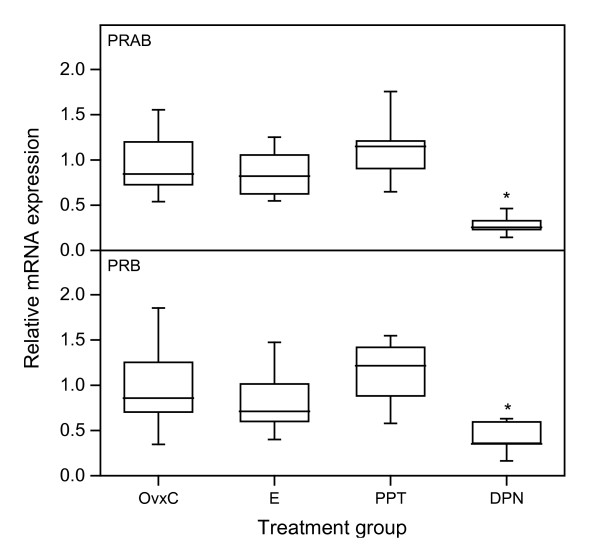
**Real-time PCR experiment 3**. Representative real-time PCR experiments for expression of PRAB (upper panel) and PRB (bottom panel) mRNAs in uterus from rats subjected to treatment with E2, PPT and DPN. The values of relative expression of target genes were normalized against RPLP0 and displayed in arbitrary units. The "box-and-whisker plot" represents the median value with 50% of all data falling within the box. The whiskers extend to the 5th and 95th percentiles. An asterisk indicates a significant (p < 0.05) difference compared to the OvxC group.

Expression of PRAB mRNA decreased in the DPN treated animals (Fig 8; upper panel). This decrease was also found in PRB mRNA levels (Fig 8; bottom panel). We did not find any effect of the other treatments on PR mRNA expression in this short time study (Fig 8).

## Discussion

The uteri of PR knock-out mice fail to support implantation after embryo transfer and are unresponsive to decidual stimulation [22], emphasising the essential function of progesterone stimulation for pregnancy to occur. The selective PRB knock-out mice have normal implantation and decidualization (reviewed in [23]. The selective expression of PRB in the uterus (PRA knock-outs) results in some PRB dependent proliferative activity, indicating that PRA is required to adverse some proliferative effects of the PRB protein [24]. This predicts that the relative levels of the uterine PR isoforms can be expected to play an important role in the responsiveness to progestins [25].

The level of PRAB in the uterine stroma is very low after ovx, and increases after E2 and P treatment, but not significantly if the treatment is P only. Up to 50% of the PRAB positive cells in the OvxC group are PRB positive, thus indicating that there are cells containing both PRA and PRB in the OvxC group. Estradiol treatment the last 24 hrs before sacrifice increased the PRB level, while P treatment on its own or the last 24 hrs did not cause a significant increase in PRB positive cells. In LE the amount of PRAB positive cells are high and they are all positive also for PRB. Estradiol treatment the last 24 hrs before sacrifice decreased both PRAB and PRB levels significantly. Changes in PRB levels did not differ from PRAB, thus PRB seems to be the dominating PR subtype in LE.

In the myometrium PR levels were low in the OvxC group, E2 the last 24 hrs prior to sacrifice increased PR levels, while P treatment had no effect, or when given after 24 hrs of E2 treatment, reduced the effect from E2 stimulation. Thus, PRAB and PRB immunohistochemistry showed similar results. From the experiment with E2 and/or P treatment only stroma seems to express PRA in cells not expressing PRB, since in this compartment differences between PRAB and PRB immunostaining are apparent. In mice, it has been shown before that PRs are expressed in epithelial, stromal and myometrial cells of the uterus and their location and temporal variation within the tissue are regulated by both estrogens and progesterone [9,25,26].

In pregnant rats the decidua was strongly stained for PRAB, while PRB was less strong, i.e. PRA seems to be highly expressed in the decidua. This is in agreement with the results from PRAB knock-out mice, where no implantation and no decidual stimulation were obtained [22], while PRB knock-outs (i.e. PRA intact) have normal implantation and decidualization [23]. Thus, PRA is the isoform most important for an implantation to occur. An alternative explanation to our results would be that the third isoforms of PR described, and named C [27] would be the dominant form in the decidua [28]. It has been shown that PRC is present in the cytosol, which is different to PRA and PRB which are nuclear [29]. The immunostaining of PRAB in the uteri and decidua is strictly nuclear, see fig 4. In the uterine stroma of the early pregnant rats PRAB immunostaining was strong, while PRB was very faintly expressed. Therefore, PRA seems also to be strongly induced in the uterine stroma during early pregnancy. The placenta was negative for both PRAB and PRB. The LE of the early pregnant rats was also negative, in agreement with the findings in the animals receiving estradiol treatment.

In immature rats both the luminal and glandular epithelia were strongly stained for PRAB and PRB, while stroma and myometrium was faintly positive. In the mature cycling animals the LE and GE are less positive for PRs while stroma and myometrium are much stronger stained, although varying during the cycle. Dramatic changes in PR expression have been shown during the estrous cycle and in early pregnancy [8]. The PR mRNA levels do not vary in the rat uterus during the estrous cycle, suggesting that the expression of PR is primarily regulated post-translationally and/or translationally [30].

The doses of PPT and DPN were decided by comparing data from previous articles on mice and rats [31-34]. They showed that PPT, similarly to E2, increases uterine weight, while no effect was found after DPN treatment. Another group showed that DPN in this concentration has anxiolytic properties in the rat [33]. Thus, the doses that we used in this short time study have previously been shown to be effective. DPN decreased the uterine PR mRNA level as compared to controls [31], which is in agreement with our present finding in this short time study. In addition, we found that DPN also decreased the PRAB immunostaining of GE, which indicate that the decrease in PR mRNA levels could be most pronounced, or selective, in the GE. No effect on the mRNA levels was found after E2 or PPT treatment. This could be caused by the effects from different cell types, since epithelium showed decreased immunostaining while stroma and myometrium had increased levels after treatment. Thus, the regulation of PR protein levels via ERα found, could be caused by translational regulation, or taking place post-translationally via regulation of other regulatory proteins. A third plausible explanation is that the ERα mediated effects on PR mRNA levels could be obscured due to the mixture of cell types in the uterine homogenate used for PCR. Further in situ hybridization studies would be informative on PR mRNA regulation by E2 and PPT in the different cell types.

It has been shown that immunohistochemical staining of uterine PR decrease in epithelial cells after E2 treatment in immature mice, in agreement with our results on ovx rats, and those from ERβ knock-out animals [35]. Thus, E2 dependent down regulation of epithelial cells in the uterus would be at least partly ERβ dependent. On the other hand, results from E2 treatment of ERα knock-out mice showed that the PR protein is up-regulated in uterine epithelium, and the level is not possible to decrease by E2 treatment in the ERα knock-out mice, indicating that the low level of ERβ in uterine epithelium is not capable of substituting for the absence of ERα [26]. The elegant experiments performed by Kurita et al. on tissue cultures of stroma and epithelium from different combinations of wt and ERaKO suggest that E2 induced down-regulation of epithelial PR in the uterus is mediated through paracrine mechanisms mediated via ERα [26]. We found though, that DPN treatment caused down regulation of the PR protein level in GE, and indeed it also decreased the PRAB and PRB mRNA levels. Therefore, epithelial PR expression (especially glandular) in the uterus is likely to be, at least partly, regulated via ERβ.

## Conclusion

The results of the present study indicate that ERα activity up-regulate PR concentrations in the stroma and myometrium, while it down-regulates the levels in epithelial cells. ERβ signalling decrease the PR mRNA levels, possibly selectively in the GE since in this structure the PR protein is also down regulated after DPN treatment. Thus, the distribution and amount of PR isoforms are depending on the hormonal milieu, the cell type within the rat uterus, as well as the subtype of ER that is activated.

## Competing interests

The author(s) declare that they have no competing interests.

## Authors' contributions

LS planned the experiments, did the animal operations and wrote the manuscript, BM and SÅ run all the analyses and gave comments on the manuscript, BM run the statistical analyses together with LS and prepared the figures, HE has been participating in the discussions about the results and the writing of the manuscript. All authors read and approved the final manuscript.
